# Development and Application of a 3D-Printed Microfluidic Sulfide-Selective Sensor for Online Monitoring of a Hydrogenotrophic Sulfidogenic Bioreactor

**DOI:** 10.3390/nano16030209

**Published:** 2026-02-06

**Authors:** David Cueto, Juan Antonio Baeza, David Gabriel, Mireia Baeza

**Affiliations:** 1 GENOCOV Research Group, Department of Chemical, Biological and Environmental Engineering, Escola d’Enginyeria, Universitat Autònoma de Barcelona, 08193 Bellaterra, Spain; davidcueto90@gmail.com (D.C.); juanantonio.baeza@uab.cat (J.A.B.); david.gabriel@uab.cat (D.G.); 2 GENOCOV Research Group, Department of Chemistry, Facultat de Ciències, Universitat Autònoma de Barcelona, 08193 Bellaterra, Spain

**Keywords:** sulfide measurement, 3D printing, sulfidogenic reactors, online monitoring, electrochemical analytical techniques

## Abstract

A sulfide online-monitoring system (S-OMS) was developed using a 3D-printed microfluidic platform to monitor sulfide in bioreactors. The S-OMS consisted of an electrochemical cell in which a microdevice was 3D-printed with co-polyester filaments and used an internal silver/silver sulfide (Ag/Ag_2_S) working electrode and a commercial, external silver/silver chloride (Ag/AgCl) reference electrode. The analytical evaluation showed a wide linear range (1.5–30,400 mg L^−1^) with repeatability and reproducibility presenting relative standard deviations of less than 5%. The S-OMS remained stable during working periods ranging from 16 h to 8 days, depending on the operation mode. Real samples from a sulfate-reducing bioreactor were used to validate the S-OMS, and the results were compared with those of a commercial sulfide ion-selective electrode (S^2−^-ISE), yielding a good linear correlation (*R*^2^ = 0.92). Moreover, a *t*-test revealed no significant statistical difference between the two analytical methods. The bioreactor operation resulted in a high sulfate reduction rate and in the accumulation of total sulfide, as measured with the S-OMS, in the bioreactor. However, the H_2_S inhibition was offset by an increase in pH and volatile suspended solids (VSS) throughout the operation. Overall, the S-OMS demonstrated robust analytical performance and operational suitability for online monitoring of sulfide in sulfide-producing bioreactors.

## 1. Introduction

Analytical methodologies span a wide range of scientific domains. They are essential to understand process behavior, decision-making, and process control [1]. In general, analytical instruments are based on various physical principles, including chromatography, spectroscopy, electrophoresis, and electrochemistry [2]. Additionally, technologies such as lab-on-a-chip (LOC), which miniaturize and automate analytical systems, have gained prominence in recent years due to their user-friendly interfaces and low application costs.

LOC platforms can be manufactured for multiple analytical techniques using 3D printing technology, which is applied in numerous fields [3,4,5,6,7]. In particular, electroanalytical methods integrated into LOC platforms have been reported in the environmental treatment sector [8]. The monitoring and control of sulfide in sulfidogenic reactors treating sulfur-rich effluents are indispensable to prevent microbial growth inhibition and toxicity to living organisms upon disposal [9]. As detailed in Table S1 (Supplementary Information), sulfide is primarily measured offline using potentiometric techniques, such as sulfide ion-selective electrodes (S^2−^-ISE) [10,11]. Other offline fluorescence and spectrophotometric sulfide detection techniques work well at low concentrations (10^−5^–10^−3^ mg S^2−^ L^−1^) [12]. Nevertheless, the main advantages of S^2−^-ISE are its fast response time and the wide measurement range [13]. Therefore, the development and application of 3D-printed analytical platforms and flow injection analysis (FIA) for real-time, in situ sulfide monitoring are promising technologies across many processes [14,15,16]. A potential application is in the integrated treatment of flue gases (SO_2_ and NO_x_) for sulfur recovery as a value-added product [17]. This process involves physical–chemical capture of SO_2_/NO_x_ gases followed by a two-step biological treatment. The first biological step consists of reducing sulfate to sulfide, either heterotrophically, i.e., in an upflow anaerobic sludge blanket reactor (UASB) fed with crude glycerol [18], or autotrophically, i.e., in a gas-lift reactor (GLR) fed with hydrogen [19]. The second biological step involves the partial oxidation of sulfide to elemental sulfur under aerobic or anoxic conditions [20,21]. The application of potentiometric sulfide measurement, mounted on a LOC platform, is promising for online monitoring of sulfide in the biological steps of the integrated flue gas treatment. In the context of monitoring and control of gaseous streams, a kind of sensor technology has been reported, including nanocomposite-based chemical sensors and piezoelectric composite transducers for gas sensing [22]. In particular, the present work focuses on potentiometric sulfide monitoring in the liquid phase to support online control of the sulfidogenic process within integrated flue gas treatment.

This work develops and uses a sulfide online monitoring system (S-OMS) in an H_2_-fed sulfidogenic bioreactor for integrated flue gas treatment. S-OMS is based on potentiometric analysis with an Ag/Ag_2_S working electrode. The research involved three steps: (1) manufacturing the S-OMS using 3D printing technology, (2) conducting analytical characterization, and (3) applying it in the sulfidogenic GLR to monitor sulfide production.

## 2. Materials and Methods

### 2.1. Reagents and Instrumentation

Sulfide stock solutions were prepared with sodium sulfide reagent (Na_2_S·9H_2_O, content ≥ 98%, ACROS ORGANICS, Castellar del Vallès, Barcelona, Spain), standardized with lead reagent (0.1 M Pb^2+^, Orion 948206, content 97–100.5%, Thermo Fisher Scientific, Waltham, MA, USA), and stored at 4 °C in an amber borosilicate glass bottle. Sulfide solutions were mixed with sulfide antioxidant buffer (SAOB) for standardization and calibration. SAOB prescription was as follows: 80 g L^−1^ of sodium hydroxide (Pharmpur, SO04201000, content 97–100.5%, Scharlab, Polinya, Barcelona, Spain), 67 g L^−1^ of ethylenediamine tetraacetic acid (EDTA, ExpertQ, ACS, AC09631000, content 99–101%, Scharlab), and 34 g L^−1^ L(+)-ascorbic acid (ExpertQ, ACS, AC05151000, ≥99.7%, Scharlab). SAOB was used as a conditioning/preservation buffer to maintain sulfide in its dissociated form (aim of NaOH), to prevent sulfide precipitation with metals (aim of EDTA), and to minimize sulfide oxidative losses (aim of ascorbic acid). SAOB was prepared weekly and used fresh for each calibration/measurement set.

The microdevice was printed with co-polyester filament (CPE, diameter: 2.85 mm, color Fabb_XT, AMPHORA, Farnell, Canal Road, Leeds, UK). An Ag/Ag_2_S working electrode and a commercial double junction Ag/AgCl reference electrode (Orion^TM^ 900100 Sure-Flow^TM^ Reference Half-Cell Electrode, Thermo Scientific) were used in the S-OMS. The working electrode was manufactured using a silver wire (diameter: 0.5 mm, purity: 99.99%, AG005150, MicroPlanet, Sant Cugat del Vallès, Barcelona, Spain). The reference electrode worked with outer (Orion 900003, 0.1 M KNO_3_) and inner (Orion 900002, 0.1 M KCl) filling solutions. An Autolab potentiostat (PGSTAT204, Metrohm Autolab B.V, Eco Chemie, Utrecht, The Netherlands) and software Nova 2.1 (Build 6159, Metrohm Autolab B.V) were used to record the electrical potential (E_c_) in the S-OMS experiments.

A commercial S^2−^-ISE (Orion^TM^, S^2−^-ISE, 9616BNWP, Thermo Scientific) was used to standardize sulfide stock solutions and to compare sulfide measurements with those obtained using the S-OMS. The S^2−^-ISE uses a KNO_3_ filling solution (B Optimum Results^TM^, Orion 900062, Thermo Scientific) and a multimeter for E_c_ recording (SB90M5 Symphony multimeter, VWR, Champaign, IL, USA).

### 2.2. Sulfide Stock Solutions and Standardization

Sulfide stock solutions were standardized weekly to quantify the total dissolved sulfide (TDS) concentration, as the hydration of sodium sulfide reagent and sulfide loss (oxidation and/or stripping) could alter the prepared concentrations. The commercial S^2−^-ISE was standardized by titrating the lead reagent in a 100-mL beaker with 25 mL of Milli-Q water (Millipore, Billerica, MA, USA) and 25 mL of SAOB. The detailed description is provided in the Supplementary Information (Section S2), as reported in the literature [23]. All sulfide stock solutions and their calibration dilutions were prepared in deionized Milli-Q water (18.0 MΩ·cm), which was oxygen-free after being bubbled with N_2_ for 10 min, and the vessels were always purged with N_2_ to minimize oxygen in the liquid and headspace.

### 2.3. Ag/Ag_2_S Electrode Fabrication

The Ag/Ag_2_S electrode was fabricated by electrodeposition as described by Pride et al. [24]: (1) the Ag/AgCl reference electrode, a Pt-counter electrode (Crison Instruments, Alella, Barcelona, Spain), and 1.5 cm of Ag wire were plugged into the Autolab potentiostat; (2) these electrodes were placed in 50 mL of a 0.1 M TDS solution (with 1 M NaOH); (3) sulfide was electro-deposited over the Ag wire by applying 2.1 V for 30 s.

### 2.4. Characterization of Ag/Ag_2_S Working Electrode

#### 2.4.1. SEM and EDX

The untreated Ag wire and the Ag/Ag_2_S working electrode were examined by scanning electron microscopy (SEM) at 5 kV to assess morphological changes. The surface elemental composition of both wires was studied using energy-dispersive X-ray spectroscopy (SEM-EDX) at 15 kV, as suggested by Pol et al. [14]. Zeiss Merlin equipment (high-resolution SEM with EDX and EBSD from Carl Zeiss Microscopy (GmbH, Oberkochen, Germany), Oxford LINCA X-Max detector from Jeol (Peabody, MA, USA)) was used for these measurements at the Microscopy Service of the Autonomous University of Barcelona.

#### 2.4.2. Calibration Procedure for Sulfide Electrodes

The potentiometric response relies on the reaction that occurs at the Ag/Ag_2_S working electrode (Equation (1)):

(1) $${\mathrm{Ag}}_{2}S_{\left(s\right)}\;+\;{2e}^{-}\;↔\;{2\mathrm{Ag}}_{\left(s\right)}\;+\;{S^{2-}}_{\left(\mathrm{aq}\right)}$$

The latter is a second-kind electrode suitable for measuring sulfide (S^2−^) [24]. Meanwhile, the reference electrode is kept at a constant electrical potential related to an internal reference solution. The electrical potential (E_c_) is associated with the sulfide concentration when ionic strength is fixed by the Nernst Equation (Equation (2)) [14]:

(2) $$E_{c}\;=\;K^{\prime }\;-\;\frac{2.303\mathrm{RT}}{\mathrm{nF}}\log\left[S^{2-}\right]$$

where the constant fraction K′ incorporates the (working and reference) electrode redox standard potentials and the term associated with the activity coefficient under a fixed ionic strength background, R is the universal gas constant (8.314 J K^−1^ mol^−1^), T is the absolute temperature, F is Faraday’s constant (96,487 C·mol^−1^), n is the number of moles of electrons exchanged (2 mol e^−^ according to Equation (1)) and [S^2−^] is the sulfide concentration.

The Nernst equation is rigorously expressed in terms of the activity of free sulfide. In practice, sulfide ISE calibrations are commonly performed using concentration because ionic strength is controlled with an ionic strength adjuster/buffer, such as a SAOB, which is approximately constant, and its contribution is absorbed into the calibration intercept, i.e., K′ in Equation (2). Additionally, samples in this study were prepared in a synthetic mineral medium (MM) and conditioned with SAOB at a fixed 1:1 ratio prior to potentiometric measurement, providing a reproducible ionic background across experiments and maintaining calibration and operation under identical chemical conditioning. Therefore, the concentration-based calibration used here represents an operational form of the activity-based relationship under controlled ionic-strength conditions.

The calibration of the sulfide electrodes involved evaluating the linear relationship between the logarithm of the TDS concentration and the E_C_ recorded with the Autolab. Then, the slope (*m*) was compared with the theoretical value (−29.09 mV dec^−1^) at the working temperature (~20 °C/293.15 K) using Equation (2). The calibration of the commercial S^2−^-ISE was performed using a successive addition method (Table S2). Meanwhile, the Ag/Ag_2_S working electrode was calibrated similarly using the Ag/AgCl reference electrode (Table S3) before assembly into the S-OMS. The S-OMS calibration consisted of sequentially adding sulfide stock solutions (Table S4). The adjusted ionic strength was used for all potentiometric measurements. A detailed description of all calibrations is found in Section S3 of the Supplementary Information.

### 2.5. Manufacturing of the S-OMS

The microdevice design was based on the literature report [14], but in our case, a commercial Ag/AgCl reference electrode was used. The layout was created in Autodesk Fusion 360 and saved as G-code. The 3D model was exported from Cura 3.2 (BCN3D Technologies, Inc., Barcelona, Spain) as an STL file for manufacturing on a Sigma R16 3D printer (BCN3D Technologies, Inc.). A hotend e3D-0.4 mm-Brass was used, with printing and plate temperatures of 255 °C and 70 °C, respectively. The printing lasted 3 h and 30 min, and the microdevice weighed ~8 g. The microdevice design and printing setup are detailed in the Supplementary Information (Figure S1 and Table S5).

The microdevice stability was evaluated by continuously flowing a strong alkaline solution, SAOB, through the S-OMS for 24 h. No leakage or visible degradation was observed, supporting the short-term chemical and mechanical resistance of the CPE-based microdevice with strong alkaline SAOB during the experimental operation. As no damage or leaking was observed, the S-OMS design and selection of CPE filament were considered a robust platform for SAOB flow. The total cost of the microdevice manufacture was estimated at 13 euros.

### 2.6. Experimental Setup

**Figure 1:**
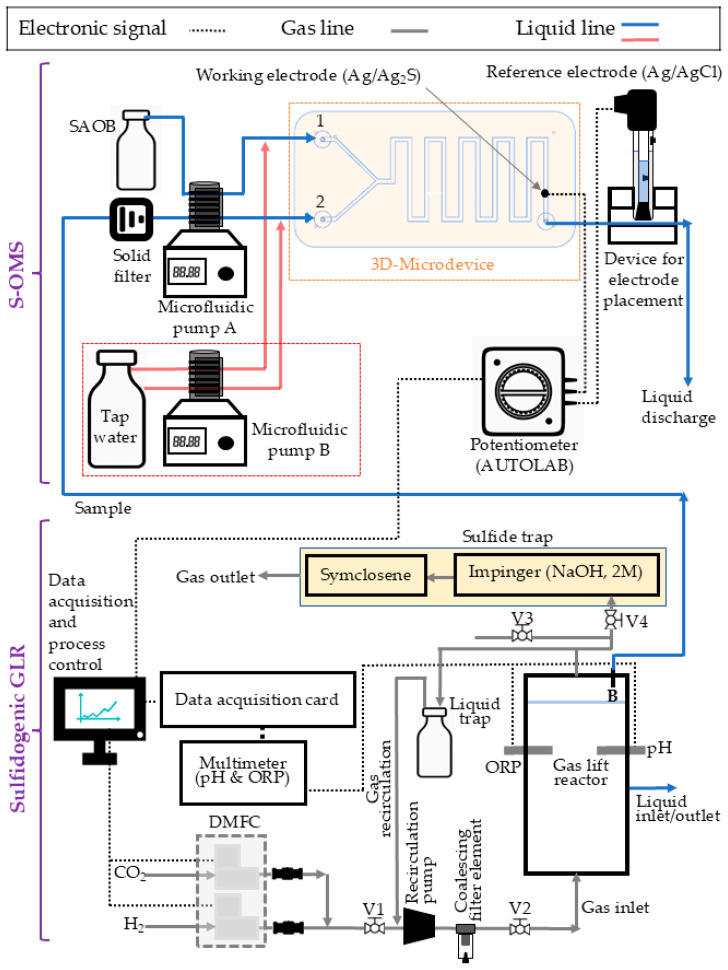
Schematic representation of the S-OMS and the sulfidogenic GLR.

The development and implementation of the S-OMS consisted of three main steps: (1) microdevice manufacture, (2) system calibration, and (3) operation for sampling the reactor to monitor TDS production. Figure 1 shows the setup of the S-OMS and sulfidogenic GLR. The S-OMS was designed to mix SAOB with the sulfide sample and to bring the resulting SAOB/sulfide stream into contact with the Ag/Ag_2_S working electrode and the Ag/AgCl reference electrode at a 1:1 ratio. The latter was installed outside the microdevice in a prefabricated methacrylate piece. SAOB and sulfide samples were pumped at 1 mL min^−1^ with a microfluidic pump (Perimax 12, SPETEC, Innofluid Co., Ltd., Shanghai, China) using Tygon tubing (Ismatec, 1.14 mm, Fisher Scientific S.L., Alcobendas, Madrid, Spain). Sulfide samples were preserved and fully dissociated with SAOB, as the Ag/Ag_2_S electrode only responds to the S^2−^ form [25,26,27]. Therefore, the sulfide concentration was accounted for as TDS.

The Autolab potentiostat was used along with Nova 2.1 software for data collection and setup. The frequency of the GLR sampling was controlled by turning microfluidic pump A on and off via custom software (ADD Control, LabWindows/CVI 2020, National Instruments, Austin, TX, USA). A washing cycle was configured in ADD Control by flowing tap water with microfluidic pump B (LabV1-II, Baoding Shenchen Precision Pump Co., Ltd., Baoding, China) to clean the microdevice channels after each measurement. This cleaning step was performed only for GLR sampling because the liquid matrix could form solid particles that could clog the microdevice or interfere with the measurement signal [28]. Additionally, GLR samples were pre-filtered with disposable inline filters (DIF-MN40, Headline Filters, Aylesford, Kent, UK) to prevent failures in the S-OMS.

The GLR was operated in sequential batch mode, and samples were taken to validate the S-OMS. Each batch cycle consisted of a reaction period, which was step I, lasting 21 to 22 h in the GLR for biological sulfate reduction, and a sedimentation period outside the reactor for sludge recovery, step II, lasting 2 h. After step I, 1.5 L of the mixed liquor was taken from the reactor for step II. Then, the supernatant (1 L) was discarded, and the settled sludge (0.5 L) was mixed with 1 L of fresh MM and returned to the reactor for a new cycle.

The reactor was operated at room temperature (~20 °C) with online monitoring of the pH (CRISON 5333) and the oxidation–reduction potential (ORP, CRISON 5353). Bronkhorst digital mass flow controllers (DMFCs) provided CO_2_ (10 mL min^−1^) and H_2_ (50 mL min^−1^) for the biological sulfate reduction. ADD Control acquired and stored pH and ORP data and configured H_2_ and CO_2_ flow rates. Part of the gas was recirculated to improve the H_2_ mass transfer rate. A 0.5-L glass bottle was installed as a liquid trap in the gas line to prevent liquid overfilling caused by clogging or foaming. The off-gas system included a sulfide trap to avoid hydrogen sulfide emissions, arranged in two stages of sulfide capture. An extended description of the sulfidogenic GLR setup is provided in the Supplementary Information (Section S5), along with a photo of the GLR setup (Figure S2).

### 2.7. Evaluation of the Analytical Quality Parameters of the S-OMS

A set of experiments was conducted to analytically evaluate the S-OMS, including the limit of detection (LOD), linear range (LR), repeatability, and reproducibility. The LOD and the LR were estimated with two independent Ag/Ag_2_S working electrodes in two phases. First, these electrodes were calibrated before being assembled in the S-OMS, with a TDS range of 0.02 to 500 mg TDS L^−1^. Then, these electrodes were installed in different S-OMS settings and calibrated over the range 0.1 to 30,400 mg TDS L^−1^.

Two sets of experiments were conducted to verify the repeatability of the S-OMS. The first consisted of measuring two sulfide solutions, in triplicate, sequentially at concentrations of 10 and 630.7 mg TDS L^−1^. The standard deviation of the E_C_ signal for each solution was used to assess analytical precision. The second experiment consisted of continuously monitoring two solutions with TDS concentrations of 2.5 and 86.0 mg L^−1^ in separate S-OMS settings, with E_C_ recorded every 0.5 s. These solutions were measured with the commercial S^2−^-ISE every 30 min to compare the two systems and assess accuracy.

Reproducibility was assessed by daily calibration of a single S-OMS with a sulfide stock solution at 17,600 mg TDS L^−1^. After each calibration, tap water and SAOB flow through the S-OMS for 2 h to simulate the extensive contact time between the electrodes and the liquid-SAOB matrix. The sensitivity (*m*) of the calibration curve, representing the analytical response, was calculated using the Nernst Equation (Equation (2)).

Additionally, the S-OMS was calibrated using TDS standards prepared in the MM matrix employed in the GLR experiments. This aimed to check the influence of the MM matrix on the potentiometric response. In that sense, two S-OMS were calibrated in triplicate, using TDS standards of 24,000 and 30,400 mg L^−1^ prepared in MM matrix.

Overall, the calibration parameters *m*, *y*_0_, the standard deviation (SD), and the relative standard deviation (RSD) were analyzed for all the S-OMS calibrations along this research to determine the analytical capacity of the S-OMS.

### 2.8. Validation of the S-OMS

The S-OMS validation is designed to sample the GLR using a unique microdevice for each batch cycle. The GLR was operated as explained in Section 2.6. The nine cycles selected for S-OMS validation corresponded to a specific experiment conducted over a year-long operation. The GLR operation consisted of a stepwise increase in the volumetric sulfate loading rate (Table S6 of the Supplementary Information). Cycle 7 was excluded from the S-OMS validation due to operational issues.

Sulfate was measured at the end of each cycle using ion chromatography (Dionex AS-AP, Thermo Scientific), which used a Dionex IonPacTM AS11-HC-4 µm column (diameter: 2 mm, height: 250 mm, Thermo Scientific) and an autosampler (Ultimate 3000 Autosampler, Thermo Fisher Scientific, Waltham, MA, USA). Potassium hydroxide (Dionex EGC 500 KOH, Thermo Scientific) was used as the column eluent at a flow rate of 0.33 mL min^−1^. Sulfate removal efficiency (Sulfate-RE), H_2_S stripping, sulfate-loading rate (SLR), and sulfate-reducing rate (SRR) were determined from Equations (S3)–(S6) (Supplementary Information) for each batch cycle to evaluate the GLR performance. Sulfate and sulfide are herein presented as sulfate-form sulfur (S-ST) and sulfide-form sulfur (TDS), respectively.

The TDS in the GLR was analyzed with the S-OMS every 2 h. This sampling consisted of switching on microfluidic pump A (Figure 1) for 5 min to measure TDS. Then, microfluidic pump B (Figure 1) was switched on for 2 min to clean the microdevice with tap water. This sampling cycle was coupled with a NOVA cycle, in which the Ec was recorded for the last 2 min while microfluidic pump A was on. This NOVA cycle avoided useless E_c_ recording, i.e., GLR samples took 3 min to flow from the GLR to the S-OMS. Moreover, the GLR was sampled at the beginning and end of each batch cycle to measure TDS with the commercial S^2−^-ISE. Linear regression and a *t*-test were used to statistically compare the results from both analytical systems.

## 3. Results and Discussion

### 3.1. Morphological Characterization of the Working Electrode

**Figure 2:**
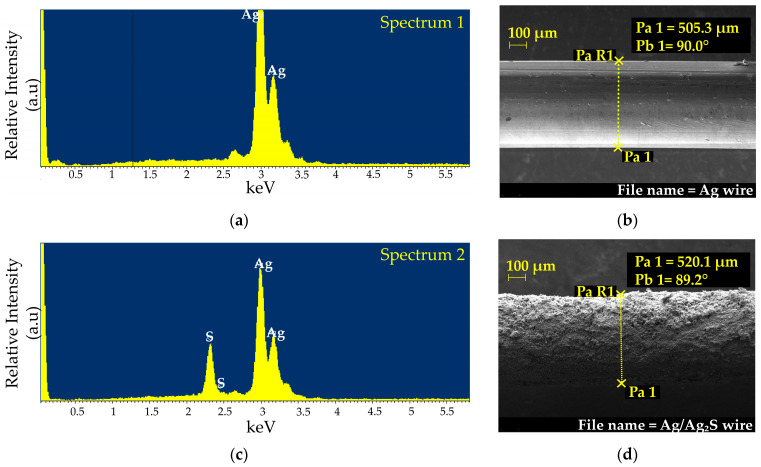
Morphological characterization before and after the electrodeposition step. (**a**) SEM micrograph and (**b**) EDX spectrum of the untreated Ag wire. (**c**) SEM micrograph and (**d**) EDX spectrum of the Ag/Ag_2_S working electrode.

The SEM and SEM-EDX analyses were performed before and after the Ag wire electrodeposition (Section 2.3), and the results are illustrated in Figure 2.

This shows the micrographs, wire diameters, and energy-dispersive spectra from SEM and SEM-EDX for both the Ag wire and the Ag/Ag_2_S working electrode. The untreated Ag wire had a smooth surface, whereas the Ag/Ag_2_S working electrode had a rough surface. The diameter difference was approximately 14.8 µm, indicating an Ag_2_S layer thickness of about 7.4 µm. EDX analysis confirmed the presence of the Ag_2_S layer. The energy dispersion spectra (keV) versus relative intensity (arbitrary units, a.u.) showed that the pure Ag wire contained only silver, while the electrodeposited wire included both Ag and S. The EDX peaks indicated 100% (in weight) of Ag for the pure Ag wire, whereas the Ag/Ag_2_S electrode had roughly 12.2% S and 87.8% Ag. This confirms that silver sulfide (Ag_2_S) forms on the working electrode surface, which is essential for potentiometric measurement.

### 3.2. Analytical Characterization of the S-OMS

Two Ag/Ag_2_S working electrodes were calibrated in the range of 0.02 to 500 mg TDS L^−1^. The LR was 0.5–500 mg TDS L^−1^. The LOD was 0.27 ± 0.05 mg TDS L^−1^, and this point was identified from the interception of the sub-Nernstian and the Nernstian slopes [29]. The linear regression (n = 18, experimental data) resulted in sensitivities (*m*) of −30.2 ± 0.5 and −28.6 ± 0.5 mV·dec^−1^, y-intercepts (*y*_0_) of −717.9 ± 0.8 and −756.9 ± 0.8 mV, and coefficients of determination (*R*^2^) of 0.996 and 0.995 for wires 1 and 2, respectively. The calibrations are depicted in Figure S3 of the Supplementary Information.

The same electrodes were calibrated in the range of 0.1 to 30,400 mg TDS L^−1^ (Table S7) after being assembled to the S-OMS (Figure S4, Supplementary Information). The LOD was 1.21 ± 0.12 mg TDS L^−1^. The LR was obtained between 1.5 and 30,400 mg TDS L^−1^, and the parameters of the linear regression (n = 6) for systems 1 and 2, respectively, were as follows: *m* = −30.02 ± 0.65 and −29.32 ± 0.91 mV·dec^−1^, *y*_0_ = −678.87 ± 1.71 and −714.55 ± 2.38 mV, and *R*^2^ = 0.998 and 0.996. These sensitivities are related to the theoretical value (*m* = 29.09 mV dec^−1^).

**Figure 3:**
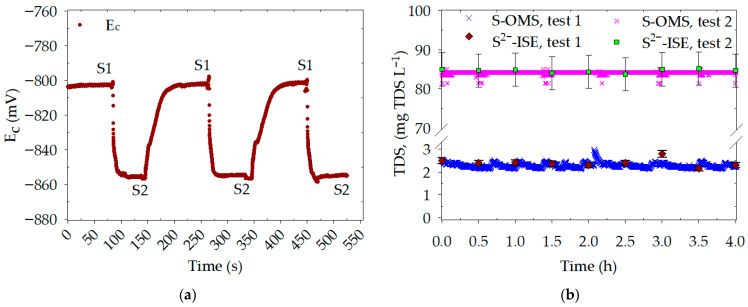
Repeatability experiment. (**a**) Electrical potential (E_c_) recorded in triplicate for 10 (S1) and 630.7 (S2) mg TDS L^−1^. (**b**) Continuous evaluation of sulfide standards at 2.3 (test 1) and 84.2 (test 2) mg TDS L^−1^ in independent S-OMS setups and measurements with a commercial S^2−^-ISE.

The repeatability experiments aimed to assess variability over a short period and were conducted in two sets. The repeatability calibrations of the S-OMS are shown in the Supplementary Information (Figures S5 and S6 and Table S8). As shown in Figure 3a, the first experiment demonstrates that standard 1 (S1) required approximately 50 s to reach a stable E_c_ after switching solutions, whereas standard 2 (S2) required approximately 20 s. Therefore, only the last 40 s of E_c_ recordings for each sampling were used to analyze the corresponding concentrations. The statistical parameters of this experiment are detailed in Table 1. The recovery percentage was calculated based on the actual concentrations—10.0 mg TDS L^−1^ for S1 and 630.7 mg TDS L^−1^ for S2—with average recovery values of 103% and 101%, respectively. The highest relative standard deviation (RSD) was 3.5% for S1 and 5.3% for S2. It has been reported that acceptable RSD for repeatability tests ranges from 4% to 6% for concentrations of 0.001% to 0.01% (*w*/*w*) (e.g., S1), and from 3% to 4% for concentrations of 0.01% to 0.1% (*w*/*w*) (e.g., S2) [30]. These results indicate good measurement accuracy in 5 of 6 cases.

In the second repeatability set, the S-OMS showed a good match with the S^2−^-ISE for both standards (Figure 3b). Standard 1 (2.3 mg TDS L^−1^) had an average concentration of 2.3 ± 0.1 mg TDS L^−1^ with an RSD of 4.4%, while the commercial method measured 2.4 ± 0.2 mg TDS L^−1^, with an RSD of 7.2% S^2−^-ISE. The RSD for the latter is affected by the deviation of data at 3 h, as only 9 samples were collected. However, around 28,800 E_c_ values were recorded by the S-OMS (collected every 0.5 s over 4 h), reducing the impact of the deviation values on the RSD. Standard 2 (84.2 mg TDS L^−1^) yielded 84.2 ± 0.2 mg TDS L^−1^ with an RSD of 0.3% for the S-OMS. This corresponded to 84.3 ± 0.7 mg TDS L^−1^ and an RSD of 0.9% for the S^2−^-ISE. In this case, the two analytical methods show a reasonable correlation.

**Figure 4:**
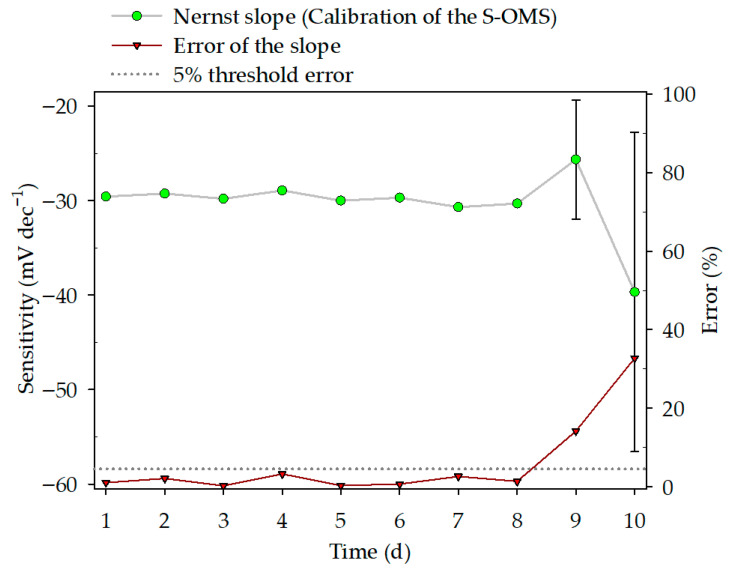
Sensitivity of the S-OMS through a reproducibility test.

Reproducibility was evaluated to assess variability over an extended period. The results are shown in Figure 4. Sensitivity is the slope (*m*) of each calibration. The error percentage was defined as the *m* deviation from the theoretical value (−29.09 mV·dec^−1^). A 5% error is plotted to visualize the calibration threshold. The sensitivity error bars (s_b_) were determined using the Excel Data Solver Tool. The daily calibration of the S-OMS and the main parameters of the linear regression are presented in the Supplementary Information (Figure S7, Table S9). The daily calibration demonstrated good Nernstian behavior over the first 8 days, as evidenced by a narrow confidence interval and errors below the 5% threshold (Figure 4). The confidence interval widened, and errors increased on days 9 (14%) and 10 (33%).

Overall, the analytical values obtained with the S-OMS can be compared to those reported in the literature, as shown in Table S1 (Supplementary Information). Pol et al. [14] and Lima et al. [16] reported LODs similar to those of the S-OMS, whereas Vallejo et al. [15] and Li et al. [11] obtained better results with FIA systems. The S-OMS has an LR similar to that of Pol et al. [14] and a response time that was remarkable compared to other works (Table S1).

The repeatability and reproducibility demonstrated the analytical reliability of the S-OMS. Nevertheless, the Nernst response and the reproducibility linear regression became deficient after 8 days, equivalent to 16 h of S-OMS operation, i.e., after electrodes were in contact with the SAOB-matrix, indicating that the Ag/Ag_2_S working electrode requires a new electrodeposition and thereby a new calibration.

Sample matrix, microbial cell debris [28], or the formation of silver complexes with EDTA [31] can diminish the sensitivity response in electroanalytical method cells. Further studies must focus on these interferences. Overall, the S-OMS working time before the need for re-electrodeposition and re-calibration could amount to 8 days of operation (Figure 4) if a measuring system is set up as detailed in Section 2.8. This recalibration period is comparable to commercial sulfide electrodes.

The S-OMS was also calibrated with TDS standards prepared in the MM matrix. This was aimed at evaluating potential interferences from the MM matrix on the potentiometric response. Two settings of the S-OMS were calibrated in triplicate to analyze variations in the sensitivity. Figure S8 and Table S10 (Supplementary Information) detail the plots and parameters for the six calibrations, which yielded *m* = −28.03 ± 1.09 mV·dec^−1^ (RDS = 3.75%) and *y*_0_ = −709.65 ± 30.62 mV (RSD = 4.32%). The RSDs for both parameters are below 5%, indicating that the MM and SAOB matrices do not interfere with the analytical response (Table S10). Selectivity in this work refers to the preferential potentiometric response of the Ag/Ag_2_S second-kind electrode to sulfide under the controlled conditioning protocol. Second-kind metallic electrodes mainly respond to their ionic counterpart and do not exhibit noticeable interference from the most common ions [25]. Ag/Ag_2_S electrodes respond solely to their specific ionic counterparts, i.e., ions capable of exchanging with the lattice or species that can reduce Ag^+^ to Ag or oxidize S^2−^ to S in Ag_2_S [14]. In this case, no evidence was found of relevant reducing or oxidizing species for Ag_2_S, and there is no lower solubility product constant (K_sp_) for silver salts.

Finally, all calibrations performed in the S-OMS were analyzed together to validate the potentiometric response. These amount to the following 29 calibrations: the two calibrations of the Ag_2_S wires (Figure S3), the two calibrations of Ag_2_S wires installed in the S-OMS (Figure S4), the calibration for the two repeatability tests (Figures S5 and S6), the eight calibrations for the reproducibility test (Figure S7), the six calibrations for the study of the MM matrix (Figure S8), and the eight calibrations for the validation experiments in the GLR (Figure S10). The parameters *m*, *y*_0_, SD, and the RSD were studied together for the 29 calibrations to describe the analytical capacity of the S-OMS. Results showed low errors based on the theoretical *m* value (−29.09 mV dec^−1^ at 20 °C) (Figure S9). All calibrations accounted for an *m* of −29.08 ± 1.16 mV dec^−1^ (RSD = 3.98%), a *y*_0_ of −744.85 ± 49.46 mV (RSD = 6.64%). These parameters demonstrated a good potentiometric response of the S-OMS, which followed the theoretical value across multiple experiments performed under different conditions. In fact, the low variability in slope and intercept across calibrations in the SAOB and MM matrices further supports the conclusions: (a) the controlled conditioning protocol provides a stable ionic background, consistent with the concentration-based operational form of Equation (2), and (b) it does not introduce noticeable interference within the investigated conditions. Yet, further studies are suggested to understand the mechanisms that affect/deteriorate the working electrode sensitivity.

### 3.3. Validation of the S-OMS in the GLR Bioreactor

The S-OMS was set to sample the GLR every two hours during each GLR cycle (see Section 2.8). In some instances, data was not recorded adequately due to software signal issues. A new S-OMS microdevice was used for each batch cycle of the GLR, based on reproducibility experiments showing a decline in analytical accuracy after about 16 h of continuous operation (see Section 3.2). As a result, a total of eight microdevices, each with a uniquely prepared Ag/Ag_2_S working electrode, were used throughout the experimental series. The calibrations for these sensors are available in the Supplementary Information (Section S11).

**Figure 5:**
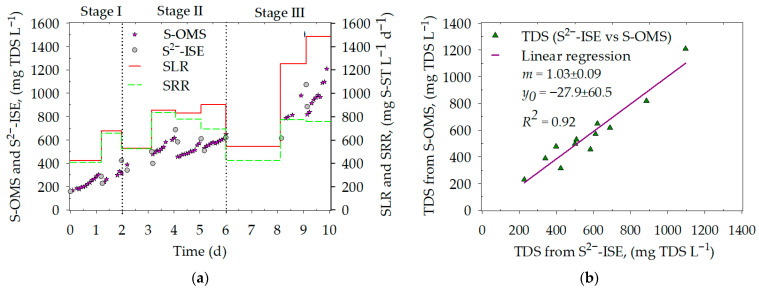
Validation of the S-OMS. (**a**) TDS data from the S-OMS (pink stars) and S^2−^-ISE (gray circles). (**b**) Linear regression test of the S-OMS vs. the S^2−^-ISE data.

Figure 5a shows the sulfide profile (S^2−^-ISE and S-OMS) and the GLR performance (sulfate-loading and sulfate-reducing rates), while Figure 5b depicts the correlation of the S^2−^-ISE and the S-OMS measurements with the linear regression parameters. The GLR operation (Figure 5a) consisted in a stepwise increase in the SLR of ~552 (stage I), ~863 (stage II), and ~1371 (stage III) mg S-ST L^−1^ d^−1^ (Table S6) with transition cycles at the beginning of stage II (cycle 3, SLR = 528 mg S-ST L^−1^ d^−1^) and III (cycle 7, SLR = 546 mg S-ST L^−1^ d^−1^). In stage I, the sulfate-RE was 96% (*w*/*w*), and sulfate was not accumulated in the reactor. In stage II, the sulfate-RE was over 97% (*w*/*w*) from cycles 3 to 5 but dropped to 77% (*w*/*w*) in cycle 6. An unexpected decrease in the SRR was observed in cycle 7, possibly due to undetected perturbations. The SRR increased to ~766 ± 11 mg S-ST L^−1^ d^−1^ with a sulfate-RE of 56 ± 8% during cycles 8 and 9.

The SRR averaged 768 ± 50 mg S-ST L^−1^ d^−1^ from cycle 4 to 9 (excluding cycle 7). Additionally, the increase in SLR (stage III) did not elevate the SRR, despite the VSS rising from 318 (cycle 1) to 610 (cycle 9) mg VSS L^−1^ and the pH rising from 7.4 to 8. This latter is associated with hydrogenotrophic sulfate-reducing activity [19]. The stagnation in the SRR could be attributed to H_2_S inhibition of sulfate-reducing microorganisms. At this point, sulfide monitoring became a key indicator of process performance. TDS increased from 287 (cycle 1) to 1095 (cycle 9) mg TDS L^−1^; however, hydrogen sulfide (H_2_S) is the sulfide form that can inhibit microbial growth [9]. Based on the pH and the dissociation constants (pka_1_ = 7.04 and pka_2_ = 11.96), H_2_S increased from 87 (cycle 1) to 108 (cycle 9) mg S-H_2_S L^−1^. H_2_S inhibition constants for sulfate-reducing microorganisms (K_i,H2S_) have been reported from 30 to 250 mg S-H_2_S L^−1^ [9], meaning that sulfide concentrations in the GLR could have halted the biological sulfate reduction. The SRR stagnated rather than decreased, which could be explained by the accumulation of solid (biomass) favored by the sequential batch operation. Therefore, this operation proved robust in overcoming sulfide inhibition in the system.

From a sulfur fate perspective, H_2_S stripping (determined from Equation (S4), Supplementary Information) saw 69 ± 16% (*w*/*w*) of the sulfate reduced. This means that a high sulfur fraction was lost through the gas outlet. Therefore, further gas-phase treatment is required at the gas outlet, either to enable further biological conversion to elemental sulfur or to prevent H_2_S discharge to the atmosphere.

The S-OMS signal originates from the Ag_2_S working electrode, whose potential is determined by the equilibrium at the Ag_2_S surface in contact with a sulfide-containing solution. The reaction is represented in Equation (1), and under equilibrium conditions, the cell potential depends on sulfide concentration according to the Nernst Equation (Equation (2)) at a fixed ionic strength. Since sulfide is a pH-dependent (H_2_S/HS^−^/S^2−^), variations in pH and ionic strength can influence the measured potential. Therefore, calibration and controlled measurement conditions are critical. The Nernstian analytical efficiency observed in this work, along with the correlation with the commercial S^2−^-ISE (Figure 5b), supports this mechanism.

Regarding the validation of the S-OMS, the results matched those of the commercial S^2−^-ISE, with both methods showing a similar trend in TDS concentrations (Figure 5a). This matching was confirmed by the linear regression parameters *m*, *y*_0_, and *R*^2^ (Figure 5b). A linear correlation is observed between the two analytical methods, as indicated by the value of *m*, which is close to 1 with a low error. In the context of real-sample validation, the GLR liquid represents a complex matrix, and the analytical result should be interpreted accordingly. Therefore, the recovery is herein reported as the agreement between methods, and accordingly, the regression slope corresponds to an average recovery of 103% over the validation range, supporting the practical applicability of the S-OMS in sulfidogenic reactor monitoring.

Regarding *R*^2^, it is a statistical parameter highly sensitive to outliers [32]; however, residual inspection (residual plot and standardized residuals) does not indicate any abnormal points. Thus, the deviations in Figure 5b reflect normal experimental variability rather than outlier-driven behavior. Data shown in Figure 5b were analyzed with a two-tailed *t*-test at a 95% confidence level. The analysis yielded a *p*-value of 0.643, a *t*-value of −0.477, and a *t*-critical value of 2.2, indicating no significant statistical difference between the two datasets. Therefore, the S-OMS system is considered equivalent to the experimental data from the commercial/crystalline membrane S^2−^-ISE.

Other flow analysis methods have been used in environmental biotechnology to identify various compounds or ions, but there are few reports specifically addressing sulfide detection [33]. Some studies have explored different configurations for measuring sulfide in the liquid phase. Spectrophotometric detection of sulfide in water has been reported by Li et al. [11] and Silva et al. [34] using FIA and sequential injection analysis (SIA), respectively. Both works reported shorter LR than in our research (Table S1). Recently, monitoring bioreactors using a 3D-printed microdevice has been evaluated for pH control in a bioelectrochemical cell, showing behavior similar to our S-OMS, with a good analytical response validated in a real process, but with a short operational lifetime due to loss of analytical sensitivity [35]. These limitations are common in 3D-printed microfluidic technologies. As comprehensively reviewed by Pan et al. [36], the integration of 3D-printed manufacturing with electrochemical sensing represents a rapidly evolving field, with promises for environmental monitoring applications where customization and cost-effectiveness are paramount, and particularly, 3D-printed electrochemical sensors have demonstrated feasibility across diverse analytical targets, including heavy metals, organic pollutants, and nutrient indicators. However, significant challenges remain in achieving detection limits suitable for direct analysis of real samples and the post-printing modifications.

Commercial S^2−^-ISE has been used for most sulfide flow analysis systems (Table S1). Villa-Gomez et al. [10] studied a sulfate-reducing reactor with in-situ measurement of sulfide using an ion-selective electrode. They obtained deviations due to pH fluctuations and sulfide concentrations over 200 mg L^−1^. Additionally, results similar to those in our work regarding response time and analytical response were reported using an S^2−^-ISE with crystalline membranes in a FIA system [16]. Nevertheless, the latter was not evaluated for real systems.

Sulfide-monitoring systems similar to ours have been studied in bioreactors for biogas treatment (Table S1). Redondo et al. [27] and Montebello et al. [26] implemented FIA systems using crystalline membrane S^2−^-ISE. The former reported a shorter LR (0.96 to 3200 mg TDS L^−1^), a similar response time, and a lower LOD than in our research. The latter did not report an LR, but it has a higher response time that hinders its application in loop control and a lower LOD than ours. Real-time and on-site monitoring of sulfide was evaluated by Liu et al. [37] using a gas-stripping module.

Herein, a modification of the Pol et al. [14] method was proposed, resulting in similar analytical properties. However, we have demonstrated the robustness of the S-OMS through GLR validation experiments.

Therefore, this S-OMS is a reliable method for future use as an analytical tool in sulfidogenic reactors that need to measure across a broad TDS concentration spectrum. However, further research is recommended to enhance accuracy with real samples.

## 4. Conclusions

The S-OMS introduced in this study was produced through 3D printing using CPE material filament. The microdevice was quick and simple to produce, demonstrating robust mechanical and chemical property stability. The proposed S-OMS can measure TDS in a wide range from 1.5 to 30,400 mg TDS L^−1^. The analytical efficiency of the S-OMS followed the theoretical value (Nernst Equation) and was proven through repeatability and reproducibility experiments. The S-OMS can operate continuously for 16 h; after that, the Ag/Ag_2_S working electrode needs to be re-electrodeposited and recalibrated.

The S-OMS was validated with real samples from an H_2_-fed sulfidogenic GLR. Its practical value was demonstrated by enabling characterization of the sulfate-reducing process, quantification of H_2_S stripping, and interpretation of how the GLR sequential operation can offset the inhibitory effect of H_2_S on microbial growth. The comparison of TDS measurements from S-OMS and the commercial S^2−^-ISE showed no significant statistical difference between the two analytical systems.

Overall, the proposed S-OMS achieves analytical performance comparable to prior sulfide flow-analysis systems while offering a simpler, low-cost, and customizable 3D-printed platform with a wide operating range suitable for sulfidogenic reactor monitoring. The main limitations are the restricted lifetime during continuous use (16 h) and, therefore, the need for further optimization to improve long-term stability and reduce recalibration frequency under extended deployment. Addressing these constraints will strengthen the applicability of the S-OMS for robust, long-duration monitoring and potential process control.

## Figures and Tables

**Table 1:** It presents the results of the initial repeatability test. It includes the mean concentration for each measurement (n = 3), along with the standard deviation (SD), the relative standard deviation (RSD), and recovery values.

Standard 1	Standard Deviation (SD)	Relative Standard Deviation (RSD)	Recovery	Standard 2	Standard Deviation (SD)	Relative Standard Deviation (RSD)	Recovery
[mg TDS L^−1^]	[mg TDS L^−1^]	(%)	(%)	[mg TDS L^−1^]	[mg TDS L^−1^]	(%)	(%)
10.7	0.2	1.8	107	655.8	25.7	3.9	104
10.4	0.3	2.4	104	631.2	33.8	5.3	100.1
9.9	0.3	3.5	99	630.3	15.3	2.4	99.9

## Data Availability

The data presented in this study are available upon request from the corresponding authors. The data is not publicly available because the repository used to store it is private, provided by the university.

## Supplementary Materials

The following supporting information can be downloaded at: https://www.mdpi.com/article/10.3390/nano16030209/s1, Table S1: Literature reports of some remarkable sulfide monitoring systems; Table S2: Successive addition method for the calibration of the S^2−^-ISE; Table S3: Successive additions to calibrate the Ag/Ag_2_S working electrodes before setting them up in the S-OMS; Table S4: Dilutions of sulfide stock solution for the calibration of the S-OMS; Table S5: 3D-printing setup for microdevice manufacture; Table S6: Operating conditions of the GLR along the nine cycles for the S-OMS validation; Table S7: Sequential sulfide flowing to calibrate the Ag/Ag_2_S working electrodes after setting them up in the S-OMS; Table S8: Parameters of the linear regressions for the calibrations of the S-OMS for the second set of repeatability experiments; Table S9: Parameters for the linear regressions of each calibration of the reproducibility experiments; Table S10: Parameters for the linear regressions of each calibration performed in the MM matrix; Table S11: Parameters for the linear regressions of each calibration of the validation experiments. Figure S1: Design and dimensions of the microdevice developed in Autodesk Fusion 360; Figure S2: Photo of the experimental setup of the GLR; Figure S3: Calibrations of two Ag/Ag_2_S wires (working electrodes); Figure S4: Evaluation of the linear response range of the S-OMS calibration curves; Figure S5: Results of the S-OMS calibration for the first set of repeatability tests; Figure S6: Calibration of the S-OMS for the second set of repeatability experiments; Figure S7: Calibration of the S-OMS for the reproducibility experiments; Figure S8: Calibrations in two S-OMS using TDS standards prepared in the MM matrix; Figure S9: Analysis of all calibrations (29) performed in this research; Figure S10: Calibration of the S-OMS for the validation experiments; Section S1: Literature reports of sulfide monitoring systems; Section S2: Standardization of a sulfide stock solution; Section S3: Calibration of the commercial S^2−^-ISE and the S-OMS; Section S4: Design and 3D-printing setup for microdevice manufacture; Section S5: Setup and performance parameters of the sequential operation of the sulfidogenic GLR; Section S6: Evaluation of the detection limit and the linear range of the electrodeposited Ag/Ag_2_S electrode; Section S7: Calibrations of the S-OMS for the repeatability experiments; Section S8: Calibration of the S-OMS for the reproducibility experiments; Section S9: Calibrations of the S-OMS with TDS standards prepared in the MM matrix; Section S10: Analysis of all calibrations performed throughout this research; Section S11: Calibration of the S-OMS for experiments performed for each batch cycle in the GLR.

## Abbreviations

The following abbreviations are used in this manuscript:

CPE	Copolyester filament
E_c_	Electrical potential
EDTA	Ethylenediamine tetraacetic acid
FIA	Flow injection analysis
GLR	Gas-lift reactor
LOC	Lab on a chip
LOD	Limit of detection
LR	Linear range
*m*	Sensitivity of the calibration curve
MM	Mineral medium
RSD	Relative standard deviation
S^2−^-ISE	Sulfide ion-selective electrodes
SAOB	Sulfide antioxidant buffer
SD	Standard deviation
SEM	Scanning electron microscopy
SEM-EDX	Energy dispersive X-ray spectroscopy
SLR	Sulfate-loading rate
SRR	Sulfate-reducing rate
S-OMS	Sulfide online monitoring system
Sulfate-RE	Sulfate removal efficiency
TDS	Total dissolved sulfide
TSS	Total suspended solids
UASB	Upflow anaerobic sludge blanket
VSS	Volatile suspended solids
*y*_0_	y-intercept
