# Supplementary material for: Development and Application of a 3D-Printed Microfluidic Sulfide-Selective Sensor for Online Monitoring of a Hydrogenotrophic Sulfidogenic Bioreactor
Source: Nanomaterials (Basel). 2026 Feb 6;16(3):209. doi: 10.3390/nano16030209 (PMC12899027; doi:10.3390/nano16030209)
Supplement: Supplementary file 1 [file nanomaterials-16-00209-s001.zip › nanomaterials-4096942-supplementary.pdf]

Supplementary Information

for

Development and Application of a 3D-Printed Microfluidic Sulfide-Selective Sensor for Online Monitoring of a Hydrogenotrophic Sulfidogenic Bioreactor

David Cueto [1], Juan Antonio Baeza [1], David Gabriel [1] and Mireia Baeza [2,*]

1 GENOCOV Research Group, Department of Chemical, Biological and Environmental Engineering, Escola d'Enginyeria, Universitat Autònoma de Barcelona, 08193 Bellaterra, Spain; davidcueto90@gmail.com (D.C.); juanantonio.baeza@uab.cat (J.A.B.); david.gabriel@uab.cat (D.G.)
2 GENOCOV Research Group, Department of Chemistry, Facultat de Ciències, Universitat Autònoma de Barcelona, 08193 Bellaterra, Spain

* Correspondence: mariadelmar.baeza@uab.cat

S1. Literature reports of sulfide monitoring systems

Table S1. Literature reports of some remarkable sulfide monitoring systems.

Sulfide analytical system	Limit of detection (LOD) [mg TDS L^{-1}]	Linear range (LR) [mg TDS L^{-1}]	Response time [s]	Reference
3D-printed sulfide electrode (online monitoring)	0.96	3.2 - 3.2·10^{4}	8	[14]
Sulfide selective electrode (FIA)	0.03	0.1 - 30	240	[15]
Sulfide selective electrode (FIA)	0.32	0.32 - (not reported)	30	[16]
3D-printed fluorescent platform (*in situ*)	-	3.2·10^{-3} - 0.16	1200	[5]
Sulfide selective electrode (online monitoring)	0.003	0.003 - 3200	10	[10]
Sulfide selective electrode (FIA)	0.48±0.29	Not reported	90	[26]
Sulfide selective electrode (FIA)	0.61	0.96 – 3,200	10	[27]
Spectrophotometric methylene blue (FIA)	1.8·10^{-5}	6.1·10^{-5} - 4.8·10^{-3}	600	[11]
Spectrophotometric methylene blue (SIA)	0.04	0.17 - 1.0	95	[34]
3D-printed microfluidic sulfide-selective sensor (online monitoring)	1.21±.0.12	1.5 - 3·10^{4}	20 - 50	This work

S2. Standardization of a sulfide stock solution

The sulfide stock solution was prepared by adding 1.6 g of the reagent to 50 mL of distilled water. The standardization was performed by titrating the 0.1 M Pb^{2+} standard in a 0.1-liter beaker containing 30 mL of SAOB and 1 mL of the sulfide-stock solution. The precipitation reaction proceeds according to Equation S1. The titration was performed while the E_c was monitored using a commercial S^{2-}-ISE connected to a Symphony multimeter. The Pb^{2+} standard was added stepwise in 0.05 mL increments using a 10 mL burette (A-6054 AFORA 10 mL ± 0.02 mL). The total sulfide consumption is determined when a sharp increase in E_c takes place, *i.e.*, the highest slope between the rise in E_c and the added volume ($\Delta Ec/\Delta V$). The sulfide concentration is then estimated from Equation S2, where V_t represents the total lead volume titrated, and V_s the sulfide volume added (1 mL). The TDS concentration is given in molarity (M) and is converted to mg L^{-1} by multiplying by 32,000.

$$S^{2-} + Pb^{2+} \rightarrow PbS\downarrow \qquad \text{Equation S1}$$

$$[S^{2-}] = 0.1[M\ Pb^{2+}]\frac{V_t[ml]}{V_S[ml]} \qquad \text{Equation S2}$$

S3. Calibration of the commercial S^{2-}-ISE and the S-OMS

Calibration of the commercial S^{2-}-ISE

Calibration was performed by adding successive volumes of the sulfide stock solution, as outlined in Table S2, resulting in the TDS concentration shown in the table. A linear regression of log [TDS] versus the recorded E_c was used for further measurements.

Table S2. Successive addition method for the calibration of the S^{2-}-ISE.

Addition of the sulfide stock solution [μL]	[TDS] [mg TDS L^{-1}]	log [TDS]	E_c [mV]
10	6.4	0.81	
10	12.8	1.11	
20	25.6	1.41	
50	57.5	1.76	
100	121.1	2.08	
150	216.1	2.33	
150	310.6	2.49	
200	435.6	2.64	
200	559.6	2.75	
250	713.3	2.85	
250	865.5	2.94	
500	1,165.5	3.07	
500	1,459.8	3.16	

*The addition (first column) of a sulfide stock solution in a 50 mL solution (25 mL Milli-Q water + 25 mL SAOB) and the resulting concentration (column 2), together with the logarithm of this concentration (column 3), are computed. The read E_c is recorded in column 4 for a further linear regression of the logarithm [TDS] vs. E_c.

Calibration of the Ag/Ag_2S working electrode before assembling the S-OMS

This calibration employed a successive addition method, as indicated in Table S3. The additions were performed using the solutions detailed in the first column, which were prepared from a sulfide stock solution of 24,000 mg TDS L^{-1} (solution 1 in Table S3).

Table S3. Successive additions to calibrate the Ag/Ag_2S working electrodes before setting them up in the S-OMS.

Solutions	Addition [µL]	TDS concentration [mg L^{-1}]
4. [24 mg TDS L^{-1}]	25	0.02
	25	0.05
	25	0.07
	25	0.10
	50	0.14
	100	0.24
3. [240 mg TDS L^{-1}]	25	0.47
	25	0.71
	25	0.95
	50	1.42
	100	2.36
2. [2,400 mg TDS L^{-1}]	25	4.71
	25	7.05
	25	9.39
	50	14.1
	100	23.3
1. [24,000 mg TDS L^{-1}]	25	46.6
	25	69.9
	25	93.1
	50	139.4
	100	231.4
	100	322.8
	100	413.4
	100	503.3

Calibration of the S-OMS

The S-OMS was calibrated with the sulfide solutions shown in Table S4. The calibration consisted of sequentially flowing these solutions and continuously flowing SAOB while E_c was recorded. The mean E_C was related to each solution concentration for calibration purposes. In some cases, dilutions lower than number 5 (Table S4) were prepared.

Table S4. Dilutions of sulfide stock solution for the calibration of the S-OMS.

**Dilution**	**Dilution factor respecting the sulfide stock solution**	**Preparation**	
		From dilution	O_2-free water
1	-	-	-
2	[10^{-1}]	1 ml of dilution 1	9 ml
3	[10^{-2}]	1 ml of dilution 2	9 ml
4	[10^{-3}]	1 ml of dilution 3	9 ml
5	[10^{-4}]	1 ml of dilution 4	9 ml

S4. Design and 3D-printing setup for microdevice manufacture

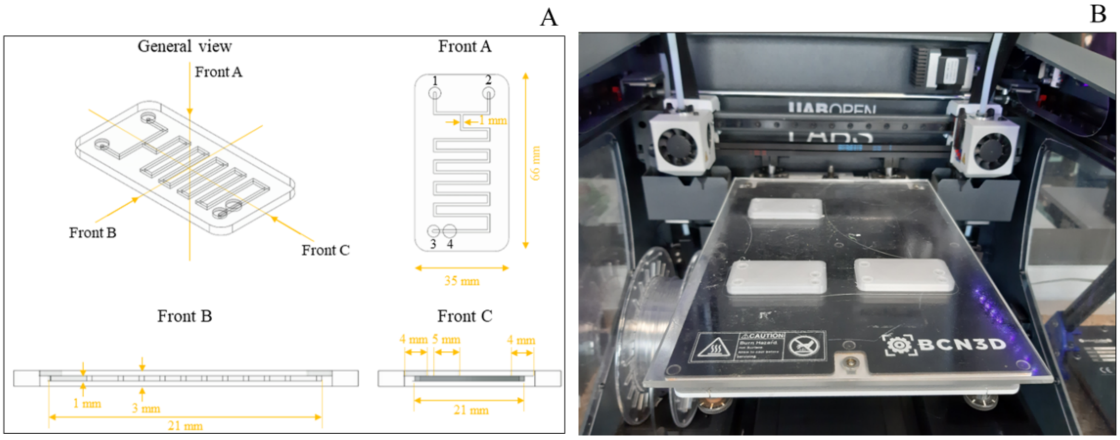

Figure S1. Design and dimensions of the microdevice developed in Autodesk Fusion 360. (A) S-OMS. (B) Photo of the printing process.

The number labels on the front of A represent the SAOB inlet (1), the sample inlet (2), the outlet of the resulting SAOB/sample solution (3), and the placement of the Ag/Ag_2S working electrode (4).

Table S5. 3D-printing setup for microdevice manufacture.

Printer	-	Sigma R16 (BCN3D Technologies, Inc)
Hotend	-	e3D-0.4mm-Brass
Layer height	[mm]	0.05
Shell		
Wall thickness	[mm]	1
Top/Bottom Thickness	[mm]	0.2
Top/Bottom Thickness		Zig Zag
Infill		
Infill density	[%]	100
Infill pattern		Grid
Print infill every	Per layer	1
Material		
Printing temperature	[°C]	255
Printing temperature, initial layer	[°C]	235
Plate temperature	[°C]	70
Filament diameter	[mm]	2.85
Flow	[%]	100
Speed		
Print speed	[mm s^{-1}]	50
Wall speed	[mm s^{-1}]	15
Initial layer speed	[mm s^{-1}]	15
Building plate adhesion		
Type		Skirt
Skirt distance	[mm]	3
Skirt minimum length	[mm]	500
Other information		
Total layers	-	58
Printing duration	[hh:mm]	03:30
Microdevice Weight	[g]	~8

S5. Setup and performance parameters of the sequential operation of the sulfidogenic GLR

The gas outlet of the GLR included a sulfide trap to avoid hydrogen sulfide emissions and was arranged in two sulfide-capture steps. The first setup consisted of an impinger in which sulfide was bubbled into a 2 M NaOH solution, and the second setup comprised a plastic bottle filled with a symclosene-based (Trichloroisocyanuric acid: $C_3Cl_3N_3O_3$) and granular solid through which the gas had to pass. The recirculation and inlet gas lines were connected to a peristaltic pump (Masterflex, model 77200-60), which in turn was connected to the GLR gas inlet.

- The Sulfate-RE was determined from the sulfate consumption per batch as described in Equation S3. The H_2S-stripping per cycle was determined from Equation S4, and it is equivalent to the arithmetic difference between the sulfate consumption, Δ[S-SO_4^{2-}], and the TDS increase, Δ[S-TDS].
- The sulfate and TDS concentrations are herein, and along the whole results, represented in the equivalent sulfur concentration, S-SO_4^{2-} and S-TDS, so that Equation S4 could be valid for the H_2S stripping determination.

- Sulfate-RE, H_2S-stripping, SLR, and SRR are determined from Equations S3 to S6, where the parameters are as follows: $[S\text{-}ST]_{to}$ represents the sulfate-form sulfur at the beginning of a cycle, $[S\text{-}ST]_{end}$ represents the sulfate-form sulfur at the end of a cycle, $\Delta[S\text{-}ST]$ represents the consumption of sulfate-form sulfur in a cycle, $\Delta[S\text{-}TDS]$ represents the accumulation of sulfide-form sulfur in a cycle, and t_c is the cycle duration.

$$\text{Sulfate-RE} = 100\frac{[\text{S-ST}]_{\text{to}}\text{-}[\text{S-ST}]_{\text{end}}}{[\text{S-ST}]_{\text{to}}}, [\%] \qquad \text{Equation S3}$$

$$\text{H}_2\text{S-stripping} = 100\frac{\Delta[\text{S-ST}]\text{-}\Delta[\text{S-TDS}]}{\Delta[\text{S-ST}]}, [\%] \qquad \text{Equation S4}$$

$$\text{SLR} = \frac{[\text{S-ST}]_{\text{to}}}{\text{t}_\text{c}} \qquad \text{Equation S5}$$

$$\text{SRR} = \frac{[\text{S-ST}]_{\text{to}}\text{-}[\text{S-ST}]_{\text{end}}}{\text{t}_\text{c}} \qquad \text{Equation S6}$$

Solids (TSS and VSS) were measured in duplicate according to the standard methods, where aliquots of 10 ml were filtered with glass fiber filters (MF-Millipore™ hydrophilic glass fiber Membrane, 0.7 μm pore size, 47 mm diameter, Merck) in a filtration assembly connected to a vacuum pump, before and after use. Filters were dried in a stove (UF 75, Memmert) at 105 °C and weighed with an analytical balance. Afterwards, the filters were heated in a muffle furnace (Model: 12PR/300 series B, Hobersal) at 550 °C to drive off volatile solids and finally weighed.

Table S6. Operating conditions of the GLR along the nine cycles for the S-OMS validation.

Stage number	Cycle	Time	Sulfate in the MM	SLR
		[d]	[mg S-SO_4^{2-} L^{-1}]	[mg S-SO_4^{2-} L^{-1} d^{-1}]
I (day 0-2)	1	1.2	3,375	421
	2	0.7	3,375	681
II (day 2-6)	3	0.9	3,375	528
	4	0.9	5,063	857
	5	0.9	5,063	829
	6	0.8	5,063	904
III (day 6-10)	7	2.0	6,750	546
	8	1.0	6,750	1,254
	9	0.9	6,750	1,488

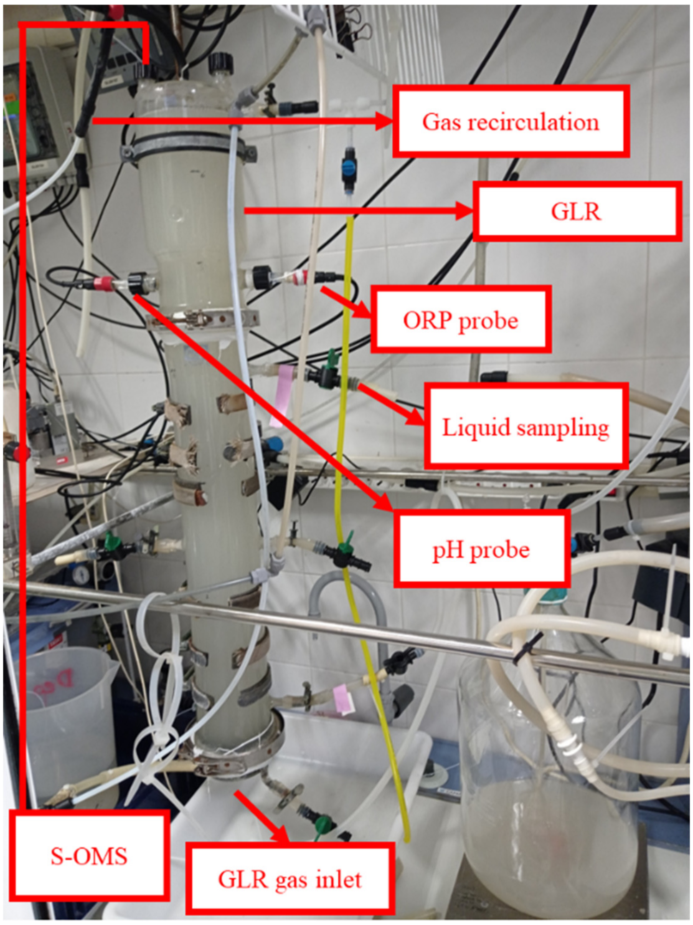

Figure S2. Photo of the experimental setup of the GLR.

S6. Evaluation of the detection limit and the linear range of the electrodeposited Ag/Ag_2S electrode

The results are shown in Figure S3, where the logarithm of the TDS concentration is plotted against the Ec (mV) for two Ag/Ag_2S electrodes.

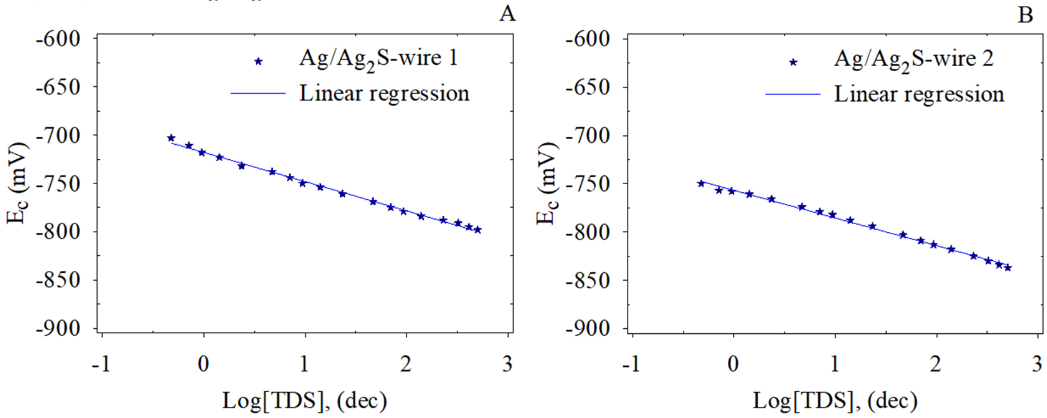

Figure S3. Calibrations of two Ag/Ag_2S wires (working electrodes).

Table S7. Sequential sulfide flowing to calibrate the Ag/Ag_2S working electrodes after setting them up in the S-OMS.

TDS concentration	Log [TDS]
[mg L^{-1}]	
0.1	-0.92
0.6	-0.22
1.5	0.18
3	0.48
30.4	1.48
304	2.48
3,040	3.48
30,400	4.48

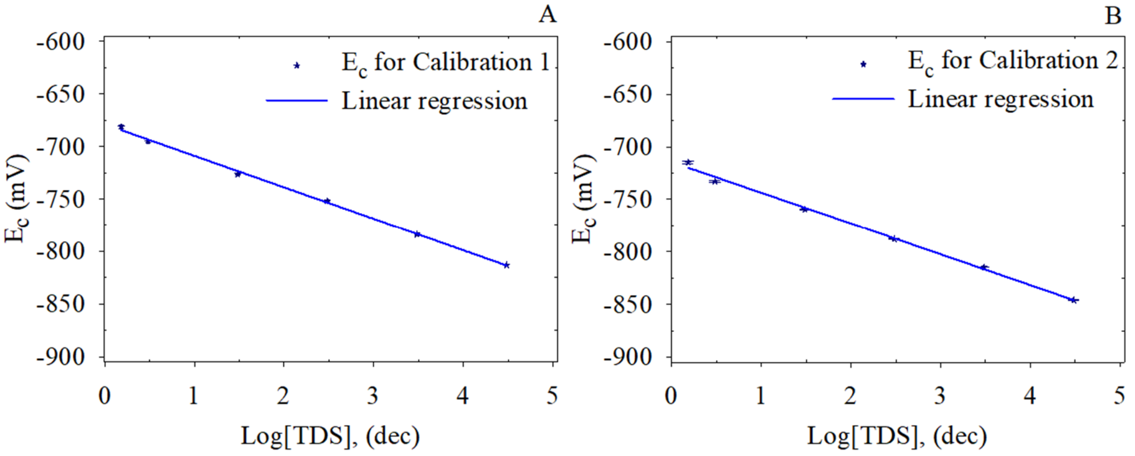

Figure S4. Evaluation of the linear response range of the S-OMS calibration curves. It was done for two independent settings, A) and B).

S7. Calibrations of the S-OMS for the repeatability experiments

Figure S5 shows the calibration performed for the first set of repeatability tests. This calibration was performed with a sulfide stock solution of 17,600 mg TDS L^{-1} and four consecutive dilutions of the order of 10 prepared from the preceding dilution.

Figure S6 shows the calibrations performed for the second set of repeatability tests. Calibrations 1 (Figure A) and 2 (Figure B) were performed for the S-OMS experiments of solutions of 2.5 and 86 mg TDS L^{-1}, respectively. The corresponding parameters for the linear regression are detailed in Table S8. This calibration was performed with a sulfide stock solution of 6,880 mg TDS L^{-1}, and three consecutive dilutions of the order of 10 prepared from the preceding dilution.

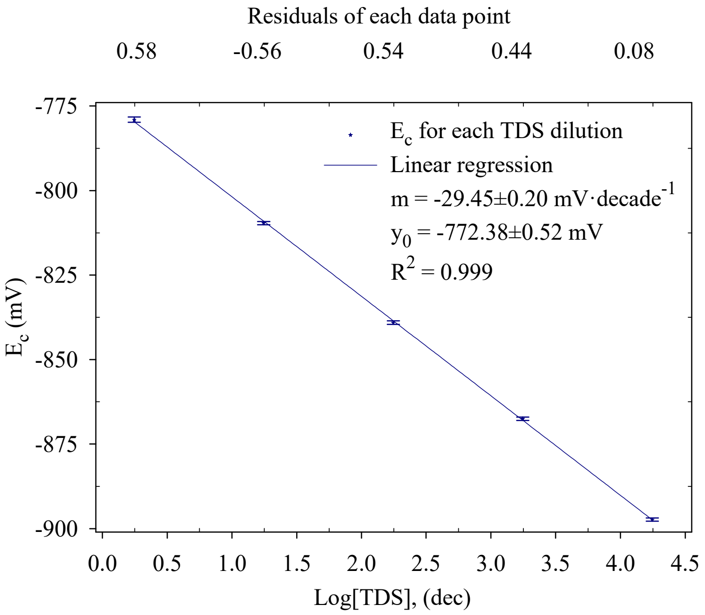

Figure S5. Results of the S-OMS calibration for the first set of repeatability tests. The logarithm of the TDS dilution is plotted against the recorded E_c.

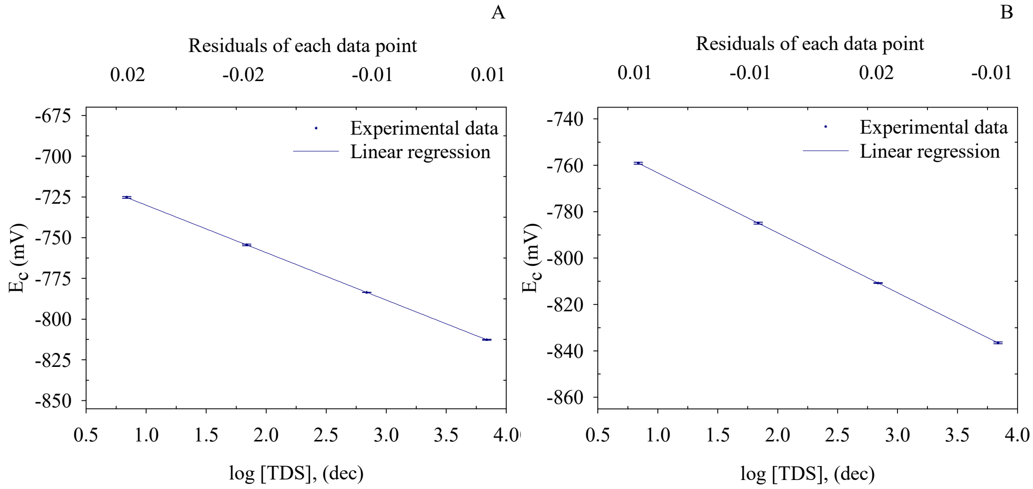

Figure S6. Calibration of the S-OMS for the second set of repeatability experiments. Each calibration was performed for two different microdevice systems.

Table S8. Parameters of the linear regressions for the calibrations of the S-OMS for the second set of repeatability experiments.

**Linear regression for each calibration**		
	CAL1	CAL2
Slope (mV·decade^{-1})	-29.10±0.02	-25.82±0.01
y-intercept (mV)	-701.0±0.02	-737.44±0.02
R^2	0.999	0.999

S8. Calibration of the S-OMS for the reproducibility experiments

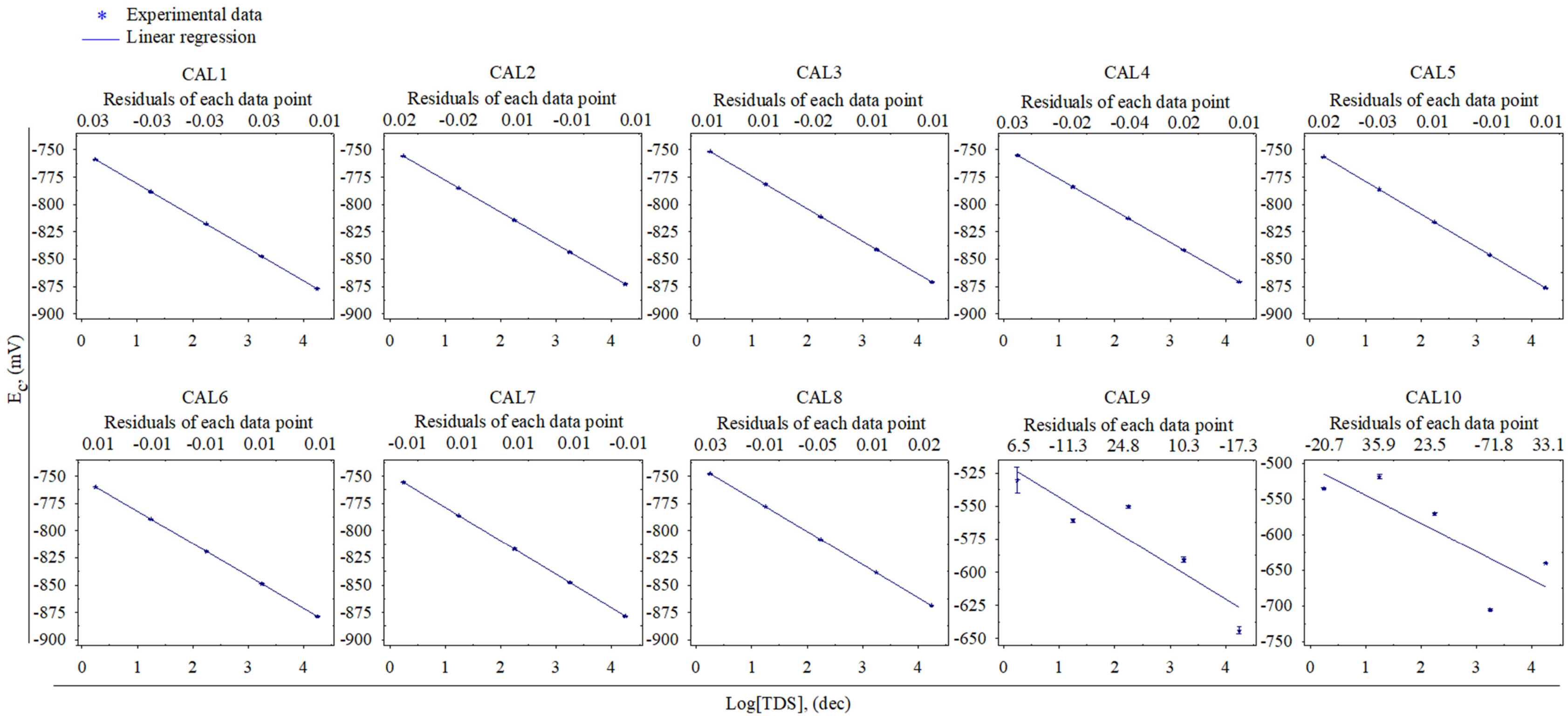

Figure S7. Calibration of the S-OMS for the reproducibility experiments. The 10 calibrations are shown in the Figures, and the corresponding parameters for the linear regression, are detailed in the table.

Table S9. Parameters for the linear regressions of each calibration of the reproducibility experiments.

Linear regression for each calibration					
	CAL1	CAL2	CAL3	CAL4	CAL5
Slope (mV·decade^{-1})	-29.58±0.01	-29.26±0.01	-29.82±0.01	-28.92±0.01	-30.00±0.01
y-intercept (mV)	-751.52±0.03	-748.57±0.02	-744.38±0.01	-747.87±0.03	-748.95±0.02
R^2	0.999	0.998	0.997	0.999	0.999
**Linear regression for each calibration**					
	CAL6	CAL7	CAL8	CAL9	CAL10
Slope (mV·decade^{-1})	-29.69±0.01	-30.69±0.01	-30.31±0.01	-25.65±6.3	-39.68±17
y-intercept (mV)	-752.50±0.01	-748.02±0.01	-740.14±0.03	-517.63±18	-504.9±45
R^2	0.989	0.999	0.999	0.847	0.649

S9. Calibrations of the S-OMS with TDS standards prepared in the MM matrix.

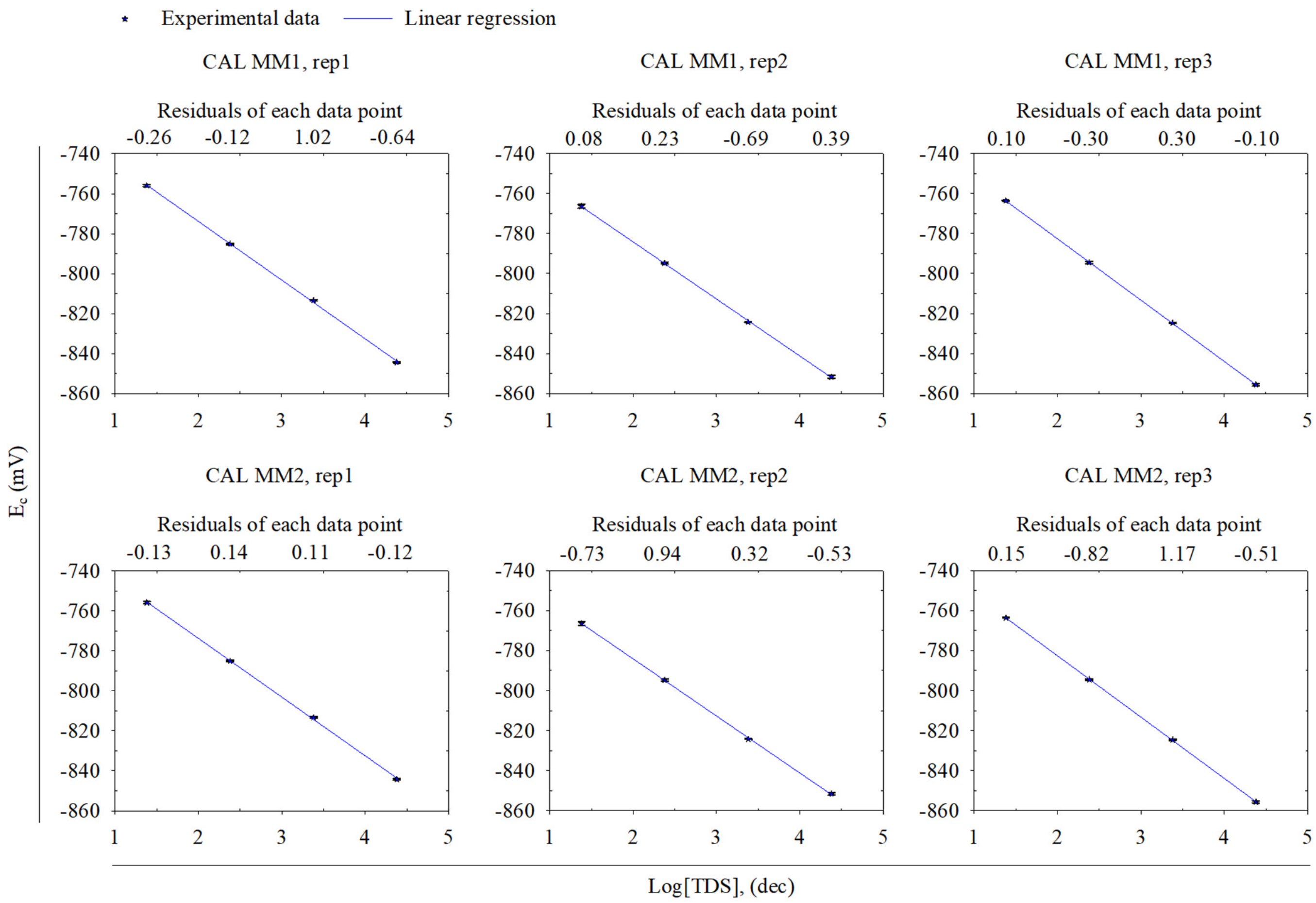

Figure S8. Calibrations in two S-OMS using TDS standards prepared in the MM matrix. MM1 and MM2 indicate the TDS standards 1 and 2, respectively, used in the calibrations. Each S-OMS was calibrated in triplicate.

Table S10. Parameters for the linear regressions of each calibration performed in the MM matrix.

**Linear regression for each calibration with the MM1 matrix**			
	rep 1	rep 2	rep 3
Slope (mV·decade^{-1})	-29.34±0.39	-28.53±0.26	-30.65±0.14
y-intercept (mV)	-715.14±1.21	-727.07±0.81	-721.27±0.44
R^2	0.999	0.999	0.999
**Linear regression for each calibration with the MM2 matrix**			
	rep 1	rep 2	rep 3
Slope (mV·decade^{-1})	-29.76±0.08	-27.77±0.43	-28.12±0.48
y-intercept (mV)	-744.93±0.25	-658.34±1.35	-691.14±1.54
R^2	0.999	0.998	0.999

S10. Analysis of all calibrations performed throughout this research

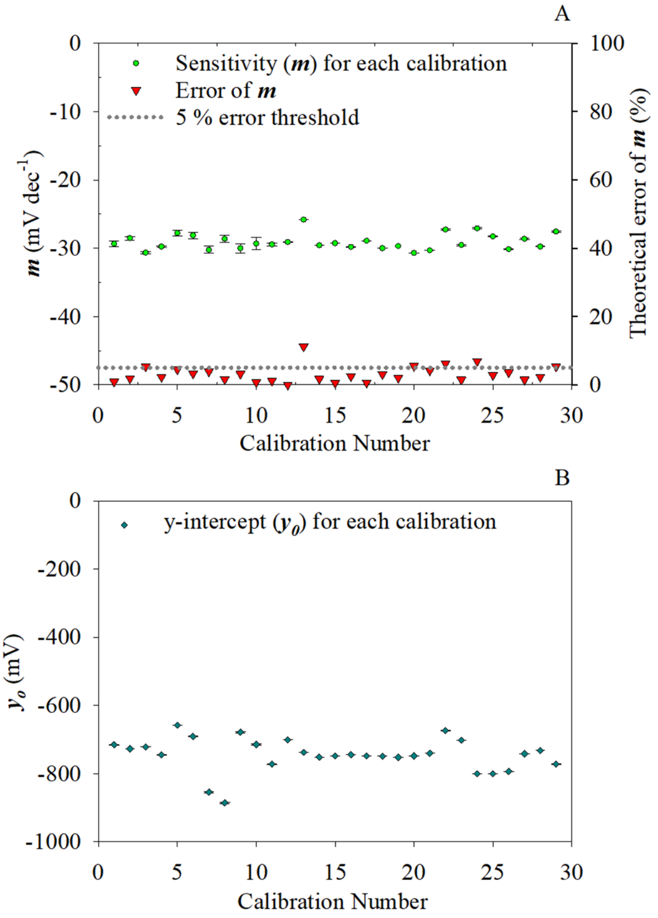

Figure S9. Analysis of all calibrations (29) performed in this research. Figure A shows the sensitivity (m) with the error of m based on the theoretical value (29.09 mV dec^{-1}), and the 5% error threshold. Figure B shows the y_o values for all calibrations.

S11. Calibration of the S-OMS for experiments performed for each batch cycle in the GLR

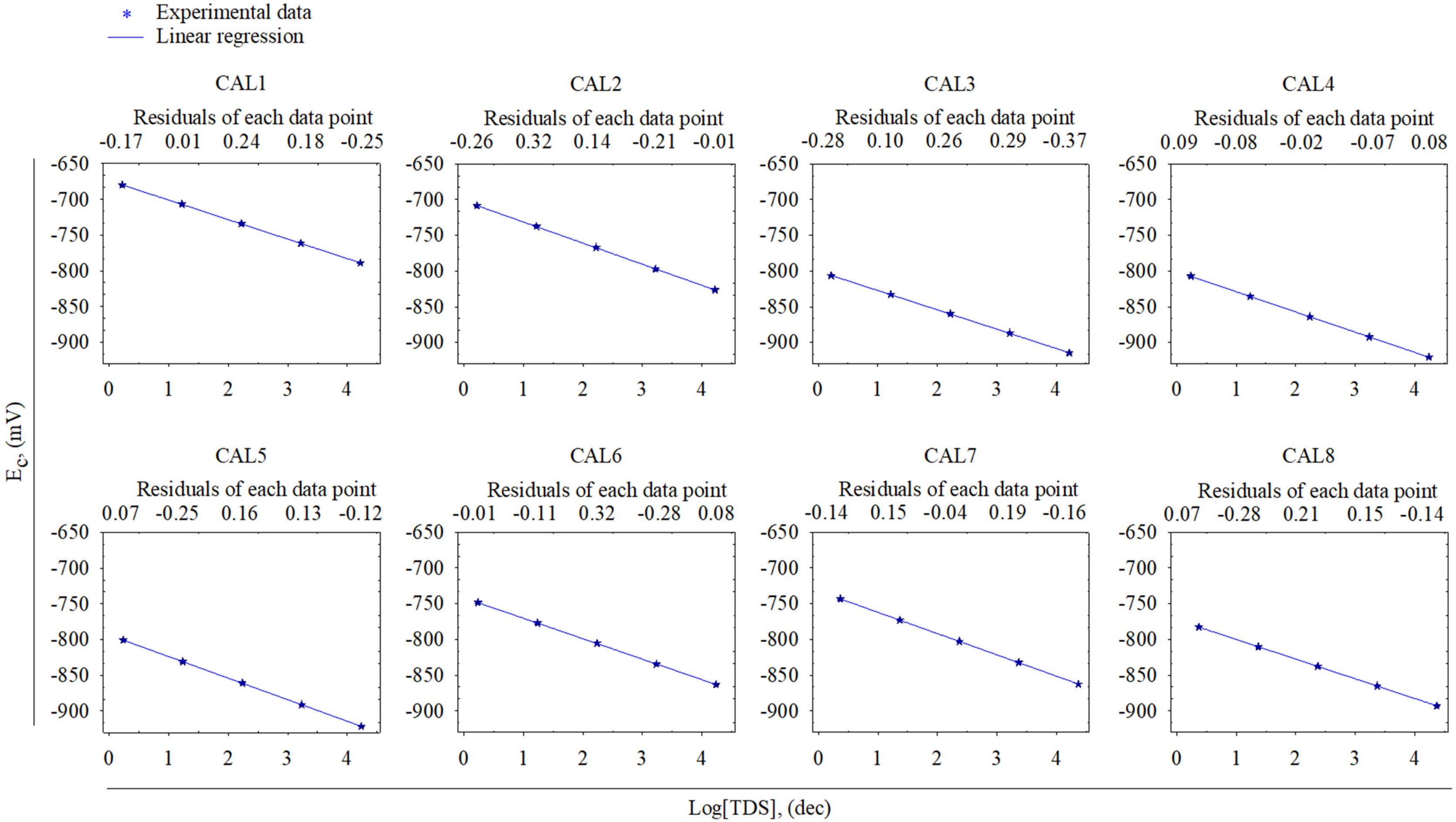

Figure S10. Calibration of the S-OMS for the validation experiments. Each calibration corresponds to the S-OMS used for each batch of the GLR operation.

Table S11. Parameters for the linear regressions of each calibration of the validation experiments.

**Linear regression for each calibration**				
	CAL1	CAL2	CAL3	CAL4
Slope (mV·decade^{-1})	-27.27±0.08	-29.53±0.09	-27.09±0.11	-28.28±0.03
y-intercept (mV)	-673.73±0.20	-701.93±0.23	-800.10±0.29	-800.7±0.08
R^2	0.999	0.997	0.995	0.999
**Linear regression for each calibration**				
	CAL5	CAL6	CAL7	CAL8
Slope (mV·decade^{-1})	-30.14±0.07	-28.64±0.08	-29.76±0.06	-27.55±0.08
y-intercept (mV)	-793.7±0.17	-741.8±0.21	-732.3±0.16	-772.5±0.20
R^2	0.999	0.998	0.999	0.999
